# Parental Perception of Terminology of Disorders of Sex Development in Western Turkey

**DOI:** 10.4274/jcrpe.0007

**Published:** 2018-07-31

**Authors:** Sibel Tiryaki, Ali Tekin, İsmail Yağmur, Samim Özen, Burcu Özbaran, Damla Gökşen, Şükran Darcan, İbrahim Ulman, Ali Avanoğlu

**Affiliations:** 1 Ege University Faculty of Medicine, Department of Pediatric Surgery, Division of Pediatric Urology, İzmir, Turkey; 2 Ege University Faculty of Medicine, Department of Pediatrics, Division of Pediatric Endocrinology, İzmir, Turkey; 3 Ege University Faculty of Medicine, Department of Child and Adolescent Psychiatry, İzmir, Turkey

**Keywords:** Disorders of sex development, intersex conditions, ambiguous genitalia, terminology

## Abstract

**Objective:**

Disorders of sex development (DSD) is a nomenclature intended to defeat the discomfort of families and patients and has found worldwide usage. The aim of this study was to address the perception and usage of terminology among the parents of DSD patients in a tertiary center in western Turkey.

**Methods:**

The records of the DSD council (multidisciplinary team where each patient with DSD is discussed) between years 2008-2015 were reviewed retrospectively. Data including details of the management process, patient characteristics and follow-up details were noted. Then inquiries reflecting parental perception about terminology were implemented during clinical visits.

**Results:**

In total, 121 patients were evaluated in monthly meetings of the DSD council and 79 inquiries were completed. Fifty-one percent of the families admitted knowing the terms DSD, ambiguous genitalia, “dubious genitals” and intersex. However, only 2% preferred using DSD, 6% intersex and 14% ambiguous genitalia. Fifty-two percent of the parents used a disease name in Latin (mostly hypospadias) addressing the disorder. The offspring of 69% of the parents who were familiar with the name “dubious genitals” were diagnosed in the neonatal period. The preferred terminology used by parents was strongly associated with the terminology used most commonly in the medical speciality their child most often attended.

**Conclusion:**

Each country has its own social norms. We suggest therefore that local committees including medical professionals, patients and families should be employed to develop proper terminology.

## What is already known on this topic?

Few studies have been conducted to explore the perspective of families on the terminology of disorders of sex development. While this terminology is in worldwide use among medical professionals’ studies have shown that the new terminology is not well accepted by affected families. All studies to date have been conducted in western countries.

## What this study adds?

This is one of the largest studies investigating parental perception of terminology about disorders of sex development (DSD). As such it reports the discontentment among parents in Turkey concerning DSD terminology. The importance of local studies reflecting linguistic and cultural differences about this complex topic are highlighted.

## Introduction

Disorders of sex development (DSD) are “congenital conditions in which development of chromosomal, gonadal, or anatomic sex is atypical” (1). Assigning an appropriate name to this condition has always been controversial (2) and medical professionals are not the only group with an interest in getting this right for everyone. The confusing nature of the disease draws the attention of health professionals, sociologists and activists. All these groups have published many papers (3,4,5,6,7), solely debating nomenclature usage. However, few have focused on patients’ families wishes and understanding. In our opinion families’ perception of the disease affects their child in two ways; by affecting their decision-making and through the environment the child will grow-up in. In the era of patient-centered medicine, questioning families’ opinion is important and necessary in order to conduct a responsible and ethical management of the condition. The aim of this study was to address the perception and use of terminology among the parents of DSD patients attending a tertiary center in western Turkey.

## Methods

In our centre the evaluation and management of DSD patients is conducted by a multidisciplinary team. Each patient with DSD is discussed at the monthly joint meetings. The department mostly involved in the management changes according to the primary diagnosis of the patient although in most cases this is pediatric endocrinology. Every critical decision influencing the management process is also taken in these meetings. Our core team consists of a pediatric urologist, a pediatric endocrinologist, a child psychiatrist, and a geneticist. Adult endocrinologists and psychiatrists, as well as pathologists, neonatologists, gynecologists and social workers are consulted when necessary.

After approval of the Ege University, Clinical Research Ethics Committee (2016; decision number: 16-2.1/1), the records of the DSD council of our institution between years 2008-2015 were reviewed. After much thought, we decided to exclude families who were presumably unaware of the terminology with regard to their child’s diagnosis (patients with Mayer-Rokitansky-Küster-Hauser (MRKH) syndrome, severe hypospadias, bilateral undescended testes). The reasoning for this was that it was felt that inclusion of these cases would risk unnecessary bias into the study. A retrospective analysis of the data including details of the management process, demographics, patient history and follow-up details was performed. Then parents were contacted to obtain their consent and to conduct inquiries focusing on the terminology the parents knew and tend to use (Appendix 1). The questionnaire consisted of closed questions and short answer open questions concerning their knowledge and preference about the terms, their first contact with the disorder and details about the management. To understand their knowledge about the terms, families were first questioned about the terms they know regarding their child’s condition. The interviewer was careful not to use any disorder names, simply calling it “the illness” during the entire interview in order not to influence their answers. At the end of the interview, if they did not recall, they were told the commonly used terms in Turkey (DSD, intersex, ambiguous genitals, dubious genitals) and asked which ones they had ever heard of. To evaluate which terms they were comfortable with, they were asked which names they use while talking to others, such as their spouses, their doctors and their relatives. They were also asked the term their doctors use and if different doctors use different names.

There were some difficulties while translating the study into English. In Turkish, there is no term as an exact translation of “intersex”. Instead, *“çift cinsiyet” *is in use (which can be translated as* double sex *in English). As* “çift cinsiyet” *is used as a translation of intersex in Turkish, implementing a meaning close to a third sex, the word* intersex *will be used in the text for ease of reading. Another interesting term in Turkish is* “kuşkulu genital yapı” *which is a distorted translation of “ambiguous genitals”. It will be used with its exact translation which is *“dubious genitals” *in the text. Our perinatologists tend to use this term, which is an acceptable catch-all phrase which avoids naming a specific diagnosis before consulting the DSD council. The term “ambiguous genitals” is included in the study as a different heading because it is used in Turkey in Latin form without being translated and therefore generates a different perception. The families mostly use it as *ambiguous* solely without understanding the meaning. In daily Turkish language, most medical terms are used in Latin, French or English, either in the original or slightly corrupted forms. Therefore, unlike parents in English-speaking countries, the word ambiguous probably appears as another disease name in Latin for them. Besides the evaluation of terminology, the parents knew and tend to use, the results were analyzed to assess the effects of different parameters on the terminology families used. These parameters included primary diagnosis, age at diagnosis (in the newborn period or later), duration of follow-up (less or more than five years), year of diagnosis (before or after 2006-year of the Chicago consensus meeting), appearance of external genitals, need for sex reassignment, need for name change, having a sibling with the disease, family history, history of admission to different hospitals and the department mostly involved in the management. 

To evaluate if the appearance of external genitals had an impact on families’ preference for the terminology, we divided patients into two groups; those who have atypical genitals and those that do not (8). Atypicality of genitals was defined as relative to the gender of rearing before reconstructive surgery. For patients reared as female, normal female and Prader Stage 1 were considered typical; Prader Stage 5 and normal male atypical. Likewise, for patients reared as males, Prader 5 and normal male were considered typical; normal female and Prader 1 atypical. Prader 2, 3 and 4 were grouped as atypical for both.

### Statistical Analysis

It was carried out using the SPSS statistical package (SPSS for windows V.16, SPSS, Chicago, IL, USA). To evaluate the effect of different variables on the terminology families used, comparisons were made using logistic regression analysis after transforming the data into dichotomous variables. Hosmer-Lemeshow goodness of fit test was used to assess model fit. A 5% type-1 error level was used to infer statistical significance.

## Results

In total 121 patients were evaluated at monthly meetings of the DSD council during the study period. Twenty-five patients with diagnoses such as MRKH, severe hypospadias and bilateral undescended testes (whose families were presumably not familiar with any of the DSD terminology) were excluded from the study. Among the rest, nine families could not be reached and four families had two affected offspring, both followed in our institution. Therefore, 79 inquiries were completed.

Median (range) age at diagnosis was 1 year (0-16 years) and 41% of the patients were diagnosed in the newborn period. Median (range) follow-up was 5 years (1-19 years). Follow up period was longer than five years in 56%. Reasons for admission at the time of diagnosis were atypical genital appearance in 55 (70%), delayed puberty in 12 (15%), inguinal hernia in seven (8%), short stature in three (4%), symptoms of salt depletion in one (1%) and incidentally during the evaluation of a syndromic child in one (1%). 

Seven patients (9%) had a history of sex reassignment and six of these also had their names changed. Four families had more than one affected child and nine parents, including these, had a family history. Forty-seven parents (41%) had been admitted to another center before referral to our institution. The majority of the parents (73%) indicated that endocrinology was the department mostly involved in the management of their children. Fifty-six (71%) children had atypical external genitalia for the gender they were reared as.

Sixty (75%) parents stated that they thought they had enough knowledge about the disease and 27 (34%) parents thought that their child knew what his/her disease was. It was noted that parents were comfortable while using the terms hypospadias or congenital adrenal hyperplasia (CAH). However, they avoided using the word “sex” during the questionnaire. An interesting observation was that some parents only said CAH when they were asked the names they know and used only CAH during the entire questionnaire. At the end, when they were asked about their knowledge of the remaining nomenclature, they first explained the pathology in the adrenal gland in detail and that the genital abnormality was secondary to it.

**Figure 1:**
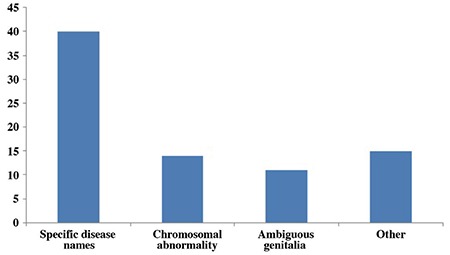
The terms parents expressed (answer to question #8)

**Figure 2:**
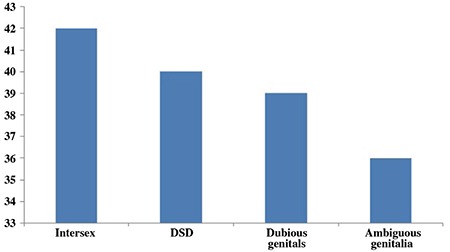
The terms parents were familiar with (answers to question #15) DSD: disorders of sex development

When asked about the terms that they could recall about the disease, 40 parents said specific disease names, mostly with Latin origin (which were hypospadias, adrenal insufficiency, testicular feminization, androgen insensitivity, CAH and 5-alpha reductase deficiency), 14 said chromosomal abnormality, 11 used the word* ambiguous*, seven referred to the name of the syndrome their child had, five said intersex and only two parents mentioned DSD. Seven parents said that they did not know any terms related to their child’s condition (Figure 1). The parents who referred to the disease as a chromosomal abnormality were the parents of children who had chromosome-gender mismatch. One parent mentioned an old Turkish word of Arabic origin *(hünsâ; khunsa) *which is not in daily use (9). When they were asked if they ever heard of the terms commonly used; 42 mentioned intersex, 40 DSD, 39 dubious genitals and 36 *ambigious *(Figure 2).

There were also questions from which we were not able to collect any comparable data. Parents were asked which terms they use when talking to their spouse, to the doctor and to relatives. The majority replied that they did not use any terms while talking to their spouse or with the doctor because everybody knew what the issue was. They also stated that they do not talk to their relatives or friends about the disease at all. They were also asked for any ideas for a new terminology, but none of the parents made any suggestions.

When the state of knowledge about each term was evaluated using independent variables, statistically significant differences were revealed between the following pairs: the term “chromosomal abnormality” or “expression of a specific disease name” and the department mostly involved in the management; the term “dubious genitals” and the diagnosis in the newborn period. 

Fourteen parents (24%) whose children were mainly followed by the endocrinology department stated the disease was a chromosomal abnormality while none of the parents who were followed by pediatric urology did (p=0.024). Expression of a specific disease name was also found relevant to the department mostly involved in the management (p=0.048). Twenty-three parents (41%) whose children were mainly followed by the endocrinology department used a specific disease name while 16 parents (80%) who were followed by pediatric urology did so.

Twenty-two of 32 (69%) parents whose children were diagnosed in the newborn period knew the term dubious genitals versus 17 of 47 (36%) who were diagnosed later (p=0.046). No statistically significant difference was found between the remaining parameters.

## Discussion

Gender is one of the major aspects of personality. Construction of a scientific terminology about a disease that interferes with gender, which is not pejorative but definitive is difficult. As Feder and Karkazis (2) perfectly describe, there is probably no terminology that can eradicate the stigma and no nomenclature that can position this group of conditions in the usual medical way. Unfortunately, parents’ perception of the terminology has a direct impact on their perception of the disease which affects how they and their child cope with the disease. 

Changing the terminology to DSD with the consensus statement in 2006 received widespread acceptance among clinicians (3). However, its perception was not the same for everyone. Linguistic, religious and cultural factors influence how the lexicon is understood. One major criticism about DSD was the disturbing effect of the word “disorder” (4). Besides the worldwide debates around it, as in German the Turkish equivalent of the word “disorder” in the phrase DSD is probably more disturbing than the English version (10). It has a meaning closer to failure or defect than disorder. It also does not have a widespread use in medical terminology. Not only the nomenclature, but also the perception and management of DSD are prone to intercultural differences (11). Some cultures do not allocate sex at birth with the belief that it can change later (12). However, gender is the major determinant of a human’s entire life in many Eastern countries. Islam has a comprehensive attitude towards DSD including prayers, obligations and gender roles in society (9). Turkey is a multicultural country where the majority of inhabitants have a social life influenced both by modern European society and Islamic beliefs. In our country, any problem related to sex will cause shame, can hinder a marriage and even affect one’s work life. Therefore, nomenclature of DSD is perhaps even more important to prevent stigmatization. Our study confirmed the importance of this issue by revealing the parents’ tendency to avoid the word “sex” during the interview.

Doctors and activists play the main roles in constituting the terminology (1,2,4,13). Affected people (children with the disease and their families) who are at the center of the arguments are mostly not a part of decision-making. Few studies have been conducted to consider the perspective of families (3,5,14,6). 

JH Davies, who is one of the proponents of the new terminology, evaluated the acceptability of the new terminology among 19 parents of children with DSD. The majority stated they preferred DSD over intersex although few found it an adequate term (3). Lin-su et al (14) interviewed a larger group (128 CAH patients, 408 parents) and stated that the majority of the patients did not like the new terminology and that it caused negative connotations. An activist, Davis, conducted interviews with patients and clinicians and argued that the patients do not like the term and the doctors’ insistence on the DSD terminology was a reassertion of their medical authority (6,7). She says the patients who embrace the new terminology are the ones who are not contented with themselves and who find themselves abnormal (7). Ellie Magritte (5), the mother of a child with DSD, used the acronym DSD when referring to the disease writing both forms (disorder/difference of sex development) and emphasized how disturbing the ambiguity was.

Our study was consistent with earlier ones showing a lack of acceptance of the term DSD by the families despite the worldwide use of it among clinicians (6,7,14,15). Half of the families admitted they knew the term and only two parents recalled it before being reminded by the interviewer. 

Lin-Su et al (14) thought that health professionals did not use the term in their daily routine with their patients. This probably has an effect but even the families who admitted knowing it did not use the term. Most of the families in our study tended to use specific disease names mostly of Latin origin. This supports Karzakis’ ideas (16) which emphasize the importance of recasting diverse diagnoses rather than keeping them as types of people whose care is directed at correcting sexual difference. Davies et al (3) and Dreger et al (17) also recommended temporary usage of the term DSD until specific diagnoses have been made. 

There is no consensus on the terminology for DSD in Turkey. International Classification of Diseases-10 still refers to the disease as hermaphroditism. Doctors use intersex or DSD while talking to each other, prefer to use* çift cinsiyet (double sex)* while talking to media, and mostly avoid using any specific term while communicating with their patients. They can also use specific disease names or some jargon such as *kuşkulu genital yapı (ambiguous genitalia)*. There is no common patient-oriented language. We have discussed the issue in our multidisciplinary council and decided to use DSD. However, our study revealed probable lack of compliance with this decision and reflected the effect of doctors’ use of terminology on parents. When asked, the parents stated that their doctors did not use any names for the disease. The findings show that the department mostly involved in the management was a factor affecting their preferences. Eighty percent of patients whose parents named the disease as “hypospadias” were mainly followed by pediatric urology, and all the parents who referred to the disease as a “chromosomal abnormality” were mainly followed by endocrinologists. This can be attributed to the need for fewer endocrinology consultations for patients without chromosome to gender mismatch but also reflects the preferences of the doctors.

We believe that specific disease names of Latin origin enhance acceptance of the subject as a medical problem, not a social one. We understand that this may not be acceptable to an adult with DSD, however families’ perception and therefore attitude towards the disease designates the adult that the child would become. None of the current terms are adequate and a terminology covering the will of both patients and families has yet to be developed.

Another interesting finding of our study is the significant difference in those knowing the term* dubious genitals (kuşkulu genital yapı)* if the condition was diagnosed as a newborn. After referral to the DSD council, families probably do not hear this term again. However, half of the families who were diagnosed in the newborn period recalled it. This not only shows the effect of doctors’ preferences but also emphasizes the persisting impact from their first contact with the disease. 

Genital atypicality, sex of rearing and the gender reassignment process were reported to cause more stigmatization of parents (8). Therefore, the effect of these variables on parents’ choice of terminology was also analyzed although no relationship was found.

Unlike other studies (3), parents stated they were satisfied with their level of knowledge about the condition. This may be due to close communication with their doctors or less expectation due to cultural motivations. DSD is a subject that is very hard to discuss in our country. Long explanations of the DSD council given by each department individually may be more than enough for the families who have never heard of and may even be trying to ignore the subject.

### Study Limitations

This study tried to evaluate the parental perception about the nomenclature of DSD; however, it was performed as a single center study in Western Turkey. Therefore, it may not reflect the opinion of all population. Also, there were semantic losses while translating the study to English. The authors tried to cover these shortcomings with a detailed methods section.

## Conclusion

Introduced with the hope of defeating the discomfort of patients and families, the term DSD does not seem to find use among the parents of patients. Parents of our DSD patients avoid using any word containing “sex” and prefer the specific disease names mostly with Latin origin instead. Their preferences were also found to be influenced by their doctors. Each country has its own social norms; therefore, local studies reflecting the linguistic and cultural differences and their uniform usage by doctors are mandatory to avoid negative connotations in the families’ minds.
